# Illness uncertainty among patients with COVID‐19 in the Mobile Cabin Hospital

**DOI:** 10.1002/nop2.924

**Published:** 2021-05-17

**Authors:** Liang Dong, Lei Chen, Shu Ding

**Affiliations:** ^1^ Department of Respiratory and Critical Care Medicine Beijing Chao‐Yang Hospital Capital Medical University Beijing China; ^2^ Renmin Hospital of Wuhan University Wuhan China; ^3^ Heart Center & Beijing Key Laboratory of Hypertension Beijing Chao‐Yang Hospital Capital Medical University Beijing China

**Keywords:** COVID‐19, illness uncertainty, infectious disease, influencing factor, nursing, pneumonia, psychological nursing

## Abstract

**Aims:**

To investigate the status and influencing factors of illness uncertainty among patients with coronavirus disease 2019 (COVID‐19) in the mobile cabin hospital.

**Design:**

A cross‐sectional study.

**Methods:**

114 patients with COVID‐19 admitted to a mobile cabin hospital in Wuhan, Hubei Province, in February 2020 were enrolled by a convenience sampling method. The Chinese version of the Mishel Illness Uncertainty Scale (MUIS) was used to assess patients’ degree of illness uncertainty, and multiple regression analysis was used to explore the influencing factors.

**Results:**

The average total score of MUIS (Chinese version) was 52.22 ± 12.51, indicating a moderate level of illness uncertainty. The dimension unpredictability turned out to have the highest mean score: 2.88 ± 0.90. The multiple stepwise regression analysis showed that female (t = 2.462, *p* = .015), monthly family income not less than RMB 10,000 (t = −2.095, *p* = .039), and disease duration of 28 days or more (t = 2.249, *p* = .027) were independent influencing factors of illness uncertainty.

**Conclusion:**

Patients with COVID‐19 are at a moderate level of illness uncertainty. Medical staffs should pay more attention to female patients, patients with lower monthly family income, patients with the prolonged disease, and take targeted interventions to help them reduce illness uncertainty.

**Impact:**

Facing the brand new and unknown infectious disease, patients confirmed of COVID‐19 suffer from immense physical and psychological stress, where illness uncertainty is a major stressor that troubles patients. The present study surveys illness uncertainty among patients with COVID‐19 in the mobile cabin hospital with results revealing a moderate level. Study results will benefit nurses in any setting where care for patients with COVID‐19 is provided, public policymakers and future researchers.

**SUMMARY STATEMENT**

1 **What is already known about this topic?**

‐ The outbreak of coronavirus disease (COVID‐19) spread rapidly, more than 300,000 confirmed cases have been reported worldwide.
‐ Facing the brand new and unknown infectious disease, patients confirmed of COVID‐19 suffer from immense physical and psychological stress, illness uncertainty is a major stressor that troubles patients.

2 **What does this paper adds?**

‐ The present study surveys illness uncertainty among patients with COVID‐19 in the mobile cabin hospital and results reveal a moderate level in our cohort.
‐ Female, monthly family income less than RMB 10,000 and duration of disease over 28 days are independent factors affecting patients’ illness uncertainty.

3 **The implications of this paper**

‐ The COVID‐19 outbreak has been declared as a public health emergency of international concern. Besides medical treatment, illness uncertainty in patients with COVID‐19 is worth more attention.
‐ These findings can be the basis for developing an intervention aimed at reduction patients’ illness uncertainty, nursing practice and research need to focus on those influencing factors or patients with specific characteristics.

## 1 INTRODUCTION

At the end of 2019, Coronavirus Disease 2019 (COVID‐19) broke out in Wuhan, Hubei Province, China, and became a major public health problem in China and the world (Huang et al., 2020). The World Health Organization (WHO) has classified it as a public health emergency of international concern (PHEIC). In order to limit the spread of the virus, the COVID‐19 Prevention and Control Commanding Center in Wuhan made a decision to build multiple mobile cabin hospitals to treat patients with mild symptoms. Facing the brand new and unknown infectious disease, patients confirmed of COVID‐19 infection suffer from immense physical and highly significant levels of psychological distress (Wang, Chudzicka‐Czupała, et al., 2020; Wang et al., 2020c; Xiong et al., 2020). Illness uncertainty is a major stressor that troubles patients, which, as defined, occurs when patients have a sense of losing control over illness‐related events and their future, and it may occur in all stages of the disease (e.g. confirmation stage, on‐treatment stage or disease‐free survival) (Mishel et al., 2018). Illness uncertainty is associated with negative psychosocial outcomes and is associated with reduced health‐related quality of life and even more severe physical symptoms (Kim et al., 2020; Parker et al., 2016; Szulczewski et al., 2017; Yang et al., 2015). The purpose of this study is to investigate the current status and influencing factors of illness uncertainty among patients with COVID‐19 and to provide the basis for related intervention studies in the future.

### 1.1 Background

COVID‐19, a new class B infectious disease mainly transmitted by respiratory droplets and close contact, is a serious viral epidemic in the 21st century that inflicted an unprecedented global impact on people's mental health. Since the outbreak of COVID‐19 in Wuhan City, Hubei Province, in late 2019, cases have been detected in 213 countries and regions. On 11 Mar 2020, the WHO declared the outbreak a global pandemic (Xiong et al., 2020). With the spread and persistence of the COVIC‐19 pandemic, the consequent psychological problems are increasingly becoming an important proposition. Many studies have shown that the COVID‐19 pandemic is associated with highly severe psychological distress. In the face of the pandemic, many people, especially COVID‐19 patients, have a series of negative emotional reactions such as anxiety and panic (Le, Dang, et al., 2020; Tee ML et al., 2020; Wang, Chudzicka‐Czupała, et al., 2020; Wang et al., 2020c; Xiong et al., 2020). The pathogenesis, incubation period and treatment of COVID‐19 are still in the exploration stage, and there are still many issues to be clarified in diagnosis, treatment and scientific cognition. The outbreak and persistence of the pandemic cause individuals to have a sense of uncertainty and uncontrollable feeling of a disease. Once the diagnosis is made, patients are uncertain as to whether there is an effective treatment, whether it can be cured, how to get through the isolation period, what will be the impact on themselves and their families, etc. The illness uncertainty puts individuals in a constant state of stress and produces anxiety, depression and fear (Hao F et al., 2020).

In 1981, Mishel defined illness uncertainty and introduced it into the nursing field. When an individual lacks the ability to determine the event related to the disease, and the disease causes the relevant stimulus event, the individual cannot make the corresponding judgement on the composition and meaning of the stimulus event, and the sense of illness uncertainty will arise. Illness uncertainty increases when a patient cannot make use of his educational background, social support or relationships with health care providers to obtain the information and knowledge he or she needs. When such events as pain, fatigue or medication‐related events occur, the lack of information increases, and so does the sense of illness uncertainty. At the same time, a high level of illness uncertainty is associated with reduced ability to process new information, predict outcomes and adapt to diagnosis (Mishel et al., 2018; Moreland & Santacroce, 2018).

Illness uncertainty has been used in studies of patients with various acute and chronic diseases, and a large number of results have shown that this cognitive appraisal of disease is associated with various negative outcomes in patients. Specifically, mood disorders are associated with high levels of illness uncertainty (Mullins et al., 2017); illness uncertainty is a predictor of depression (Zhang et al., 2018); in addition, sense of illness uncertainty is unanimously considered a malignant event (Hoth et al., 2015; Parker et al., 2016; Sharkey et al., 2018), and is believed to be related to negative psychosocial outcomes, such as emotional stress, anxiety or mental disorder (Kim et al., 2020; Szulczewski et al., 2017). Not only does it interfere with the patient's ability to seek information about the disease hinder their choice of treatment and health care (Moreland & Santacroce, 2018), but it also reduces the patient's health‐related quality of life, and is even associated with more severe physical symptoms (Guan et al., 2020; Varner et al., 2019).

In view of these negative effects of illness uncertainty, more and more researchers begin to pay attention to the level of uncertainty in patients with different diseases and try to find ways to significantly reduce illness uncertainty. Mishel's theory explains that illness uncertainty is caused by unclear disease symptoms, complex treatment and care, lack of information related to the diagnosis and severity of the disease, and unpredictable disease process and prognosis. It was also influenced by the patient's cognitive level and social support. Studies have found that the perception of illness uncertainty is influenced by many factors. Age, race, cultural concept, educational background, economic status, disease course, and whether the illness is complicated by other diseases or symptoms, etc. in the patients' demographic and clinical data have been analysed as factors influencing the perception of illness uncertainty in many studies (Parker et al., 2016).

## 2 THE STUDY

### 2.1 Aims

To investigate the status and influencing factors of the illness uncertainty among patients with COVID‐19 in the mobile cabin hospital.

### 2.2 Design

A cross‐sectional study was performed in the mobile cabin hospital, covering an area of 1,385 square metres, divided into three wards housing a total of 678 beds.

### 2.3 Participants

Using the convenience sampling method, 114 patients with COVID‐19 admitted to a mobile cabin hospital in Wuhan, Hubei Province, in February 2020 were enrolled as the research subjects. Inclusion criteria: aged 18–65 years; confirmed of COVID‐19 infection and clinically classified as mild or moderate cases in accordance with the national diagnostic and therapeutic guideline; consenting for participation in the study. Exclusion criteria: cognitive impairment or mental or psychological illness; severe visual, hearing or speech disorders.

### 2.4 Data collection

In view of the quarantine rules for COVID‐19, the survey was conducted in the form of an electronic questionnaire, and logic verification was set to improve the validity of the questionnaire. On‐the‐spot survey for this study was carried out among the patients with COVID‐19 admitted to the mobile cabin hospital, where the researchers strictly screened patients in accordance with the inclusion and exclusion criteria. The researchers instructed the patients to complete the questionnaire with unified language. The patients anonymously filled out the questionnaires by scanning a QR code.

### 2.5 Validity, reliability, and rigour

#### 2.5.1 General information questionnaire

The self‐designed general information questionnaire includes gender, age, marital status, number of children, place of residence, educational level, employment status and monthly family income, as well as the time since the onset of COVID‐19, and whether relatives and friends were infected.

#### 2.5.2 Chinese version of the Mishel uncertainty in illness scale (MUIS)

The uncertainty in illness scale was originally developed by Professor Mishel in 1981. It was revised by Ye Zengjie's team to form a Chinese version of MUIS (Ye et al., 2018), which includes three dimensions of uncertainty and a total of 20 items: ambiguity (8 items), lack of clarity (7 items) and unpredictability (5 items), with four of them being reverse‐scoring items. These items are scored 5‐point Likert scale, where 1= strongly disagree and 5= strongly agree, and the total score range is 20–100; the higher the score, the greater the uncertainty. The score is divided into three levels: low level (20–46.6), moderate level (46.7–73.3), and high level (73.3–100). The Cronbach's α of the Chinese MUIS is 0.825, and that of each dimension is 0.807–0.864.

## 3 ETHICAL CONSIDERATIONS

The participants were informed of the study aim and informed consents were obtained when the participants were recruited. Then they started to fill in and submitted the questionnaires online voluntarily.

### 3.1 Data analyses

SPSS 16.0 was used to establish a database and import the data for analysis. Count data are expressed as percentages and analysed by a chi‐square test; measurement data that conforms to the normal distribution is expressed as mean ±standard deviation and analysed by a *t*‐test, and multiple stepwise regression analysis was employed to analyse the factors affecting the illness uncertainty in patients with COVID‐19. The difference was statistically significant when *p* < .05.

## 4 RESULTS

### 4.1 General demographic characteristics

A total of 114 questionnaires were distributed in this study, with an effective recovery rate of 100%. Of the 114 patients, 51 are male and 63 female; aged 45.11 ± 11.43 years. The average number of days lapsed since the onset of COVID‐19 was 27.69 ± 10.31 days. The majority of the patients were married, taking up a total of 93 cases (81.7%). Among them, ones with spouses diagnosed with COVID‐19 accounted for 28.1%, children 12.3%, parents 28.1%, and friends 39.5%. 75.4% of the patients with COVID‐19 were most worried that the disease would affect their families; 70.2% of the patients fretted about the sequelae of the disease; 54.4% of the patients feared that the disease would worsen, which would affect their return to normal life; 32.5% of the patients were afraid that the disease would affect their work; 21.2% of the patients worried that the disease would affect the financial security of their families.

### 4.2 Current status of illness uncertainty

The total MUIS score of patients with COVID‐19 was 52.2 ± 12.5, indicating a moderate level of illness uncertainty (Table 1). We ranked the scores of each item of patients’ illness uncertainty and found that the item with the highest score was “I can't predict how long my illness (treatment) will last” (Table 2).

**TABLE 1:** MUIS scores of the 114 COVID‐19 patients (*N* = 114)

Item	Score range	Total score ($\overset{‐}{x}$±s ± s)	Mean score of Items ($\overset{‐}{x}$±s)
Total MUIS score	22–84	52.22 ± 12.51	
Ambiguity (8 items)	8–36	21.04 ± 5.09	2.62 ± 0.64
Lack of clarity (7 items)	7–29	16.79 ± 4.72	2.40 ± 0.67
Unpredictability (5 items)	5–23	14.39 ± 4.50	2.88 ± 0.90

**TABLE 2:** Top 3 scored item of MUIS (*N* = 114)

Dimension	Item	Rank	Mean (±s)
Unpredictability	8. I can't predict how long my illness (treatment) will last.	1	3.52 ± 1.09
Unpredictability	3. I am unsure if my illness is getting better or worse.	2	3.20 ± 1.21
Lack of clarity	2. I have a lot of questions without answers.	3	3.04 ± 1.23

### 4.3 Univariate analysis of COVID‐19 illness uncertainty

The general demographic data of participants was used as a grouping variable in comparing the illness uncertainty of patients with COVID‐19. The results showed that gender, monthly family income, and time since onset of the disease (t = −3.130, 2.276, −2.162, *p* < .05) were statistically significant (Table 3).

**TABLE 3:** Univariate analysis of COVID‐19 illness uncertainty (*N* = 114)

Item	Number	Percentage (%)	Score ($\bar{x}$±s)	F/t	*p*
Gender				−3.130	.002
Male	51	44.74	48.29 ± 11.63		
Female	63	55.26	55.40 ± 12.37		
Marital status				−0.165	.869
Single	21	18.42	51.81 ± 12.69		
Married	93	81.58	52.31 ± 12.53		
Place of residence				0.364	.716
City/Town	103	90.35	52.36 ± 12.26		
Country	11	9.65	50.91 ± 15.27		
Educational level			±	0.772	.546
Primary education	5	4.39	59.2 ± 12.64		
Secondary education	40	35.09	52.62 ± 12.05		
Associate degree	32	28.07	52.41 ± 11.65		
Bachelor's degree	30	26.32	51.60 ± 14.50		
Postgraduate education	7	6.14	46.57 ± 9.91		
Employment status			±	1.702	.187
Employed	68	59.65	50.53 ± 13.35		
Unemployed	19	16.67	55.89 ± 11.80		
Retired	27	23.68	53.89 ± 10.19		
Monthly family income			±	2.276	.025
<10,000	83	72.81	53.82 ± 12.58		
≥10,000	31	27.19	47.94 ± 11.43		
Time since onset				−2.162	.033
<28 days	45	39.47	49.13 ± 11.44		
≥28 days	69	60.53	54.23 ± 12.84		

### 4.4 Multivariate analysis of COVID‐19 illness uncertainty

Multivariate stepwise regression analysis was performed with the MUIS total score as the dependent variable, and three factors (gender, monthly family income and time since onset) that were statistically significant in univariate analysis and correlation analysis as independent variables. The variables that ultimately entered into the regression equation were gender, monthly family income and time since onset of COVID‐19, which were the three main factors influencing the dependent variable (Table 4).

**TABLE 4:** Multivariate analysis of COVID‐19 illness uncertainty (*N* = 114)

Factors	B	SE	Beta	t	P	95%CI	
						Lower Bound	Upper Bound
Constant	42.060	5.966		7.050	0.000	30.237	53.883
Gender	5.606	2.277	0.224	2.462	0.015	1.093	10.118
Monthly family income	−5.347	2.553	−0.191	−2.095	0.039	−10.406	−0.288
Time since onset	5.144	2.287	0.202	2.249	0.027	0.601	9.677

Note

R2 = 0.144, *F* = 6.170，*p* =.001

## 5 DISCUSSIONS

### 5.1 Illness uncertainty of patients with COVID‐19

The results of this study showed that the total MUIS score of patients with COVID‐19 was 52.2 ± 12.5, indicating a moderate level of illness uncertainty, which is consistent with studies on illness uncertainty for different diseases such as COPD, congenital heart disease, blood pressure dialysis, and fever of unknown origin at home and abroad (Hoth et al.,^2015^; Li et al., 2018; Lyu et al., 2019; Moreland & Santacroce, 2018; Yang et al., 2015). Based on Mishel's illness uncertainty theory (Mishel, 2018; Zhang, 2017), the event familiarity and congruency for COVID‐19 are at a low level since it is a new, unknown and highly infectious disease, which may lead to a high level of illness uncertainty. However, the findings of the survey do not indicate the anticipated results. Possible reasons are as follows: (a) The intensity of symptoms is a major factor in illness uncertainty (Mishel et al., 2018). According to the admission criteria of mobile cabin hospital, the patients are ones with mild symptoms. Therefore, the illness uncertainty scores did not reach a high level; (b) Social support is the main prediction factor of the illness uncertainty level. With support provided for coping with COVID‐19 at the national level, after confirmed diagnosis, patients were able to be admitted to mobile cabin hospitals in time and receive professional treatment from medical teams from provinces and cities nationwide. In addition, the treatment costs were borne by the state so that the patients had no worries, thus lowering the illness uncertainty in these patients to a certain extent; (c). A large number of patients with COVID‐19 with mild symptoms were gathered in the mobile cabin hospital. The communication among them strengthened their confidence to overcome the disease. And the active atmosphere helped the patients avoid negative emotions such as fear, anxiety and depression caused by isolation, reducing the patients' illness uncertainty to a certain extent (Parker et al., 2016; Zhang et al.,^2018^).

### 5.2 MUIS scores of patients with COVID‐19 by item

The item with the highest score is “I can't predict how long my illness (treatment) will last”, being 3.52 ± 1.09. On the one hand, since COVID‐19 is a brand‐new infectious disease, the patients barely know anything about it; on the other hand, the disease has a long course, in this study, the time since onset was 28 days or more for 69 patients, accounting for 60.53% of the total number of the respondents. The average stay time of 114 patients in the mobile cabin hospital was (13.07 ± 5.84) days. Among those, 39 people stayed more than 2 weeks (more than 14 days), taking up 34.21% of the total number. Therefore, patients assigned a higher score for this item.

The item ranking second, “I am unsure if my illness is getting better or worse,” was scored 3.20 ± 1.21. COVID‐19 is a novel, unknown and highly infectious disease. The occurrence, development and treatment of the disease are still under exploration. Patients were uncertain about how it would develop and how they would be treated, which may lead to this item with a higher score.

The item ranking third, “I have a lot of questions without answers,” was scored 3.04 ± 1.23. Confronting the unknown disease, medical staff are constantly exploring and optimizing their understanding of the disease and diagnosis and treatment plans, thus some of the disease‐related questions raised by patients may not have been fully answered. Since the ratio of medical staff in the mobile cabin hospital is generally maintained no more than a bed‐nurse ratio of 6:1 and a rotating four‐shift schedule is set up, each medical staff needs to attend quite a few patients. Besides, certain information attenuation could occur during the communication with medical staff, who wear protective clothing. Although disease treatment‐related instructions and explanations have been given to patients as much as possible, some personalized questions may not have been fully answered.

At the beginning of this global health crisis, there were differences in the information about COVID‐19 received by health care workers, community workers and the general population. Medical staff and community workers can gain a higher level of awareness and epidemic control knowledge through diversified training courses. The general population has seen a lot of negative information about COVID‐19 through the mass media, such as information related to reduce supplies of medical equipment, which has increased anxiety and illness among patients. This situation illustrates the urgent need to increase the coverage of reliable health information, as misleading information may hinder health institutions from controlling epidemics (Tran et al., 2020). High satisfaction with health information was significantly associated with lower psychological impact, illness, and anxiety or depression scores (Le, Dang, et al., 2020).

### 5.3 Main influencing factors of illness uncertainty of patients with COVID‐19

#### 5.3.1 Gender

The result of the present study of patients with COVID‐19 showed that female patients had higher levels of illness uncertainty than male patients. Mishel pointed out that as the core variable of the theory, an individual's cognitive capacities influence perception of illness‐related stimuli. The study has shown that there are significant differences in cognitive capacities between men and women (Hyde, 2014). Women are better at feelings and intuitive thinking while men are more inclined to rational analytical thinking, which can facilitate male patients' understanding of the stimuli, thus reducing their uncertainty in illness. Men and women also differ in the type and efficiency of emotion, Women prefer emotional and evasive coping styles while men tend to use problem‐solving and positive thinking strategies to cope with negative emotional events (Schmitt et al., 2017). Which also indicates that medical staff should guide patients as appropriate to help them remain neutral in accurately appraising and understanding the illness uncertainty itself.

#### 5.3.2 Monthly family income

Patients with a monthly family income more than or equal to RMB 10,000 had significantly lower MUIS scores. This finding is consistent with the other studies (Li et al.,^2019^; Ni et al.,^2018^), which revealing lower monthly family income is a positive predictor of the patients' level of illness uncertainty. The speculated reasoning behind this is that patients with lower family incomes have relatively fewer social resources and fewer channels to get information about the disease. With the instability of work and economic income, they usually have heavy family burdens. As a result, when faced with an unknown and severe disease, this group of patients is more doubts and concerns, thus showing a high level of illness uncertainty.

#### 5.3.3 Time since onset

The longer the duration of illness, the lower the patients’ sense of uncertainty (Mishel, 2018), results from studies demonstrated this point (Tian et al., 2014), saying that the increase in chronic disease diagnoses, treatments and hospitalizations help patients identify and get familiar with disease‐related events. Nevertheless, the results of the present survey showed the opposite argument. To be specific, the illness uncertainty significantly increased for those cases with 28 days or more elapsed since the onset of COVID‐19, which concurs with the results of Li (Li et al., 2018) in his studies on patients with fever of unknown reasons. The occurrence, development and treatment of chronic diseases are relatively clearer, while COVID‐19, as a novel and unexpected infectious disease, is still under exploration, to treat the disease is navigating uncharted waters, during which unexpected events have occurred, such as the patients who were cured and discharged from hospital during the infectious period yet relapsed afterwards. Due to uncertainties in the diagnosis, treatment and scientific cognition of the disease, patients with COVID‐19 are unsure about the development trend and treatments of the disease despite the time since the onset of COVID‐19 gets prolonged. Faced with the uncertainties, the more time elapsed since the onset of COVID‐19, the more worried the patients get about the treatment effect of the disease, the more uncertain the patients feel about the disease characteristics, and thus the higher illness uncertainty they have.

The result suggests that patients with the above characteristics should focus on illness, and the goal of intervention for illness is to seek a management method to reduce illness. It includes health education, information support, behavioural therapy, and cognitive‐behavioural therapy (CBT). For patients with COVID‐19, behavioural therapy can help them to combat anxiety with the use of relaxation techniques and prevent depression onset by altering the schedule of their routine activities, CBT can mitigate maladaptive coping behaviours such as avoidance, antagonistic confrontation and self‐blame by enhancing their ability to manage stress (Ho et al., 2020). Internet cognitive behavioural therapy (I‐CBT) interventions can benefit patients who become infected and receive care in isolation wards, as well as those who are isolated at home and do not have access to mental health professionals (Ho et al., 2020; Soh et al., 2020; Zhang & Ho, 2017).

## 6 CONCLUSION

The MUIS scores of patients with COVID‐19 in the mobile cabin hospital demonstrated a moderate level of illness uncertainty. The top‐scored one among the three‐dimension was *unpredictability*. The illness uncertainty was found to be positively correlated with the time since COVID‐19 onset, and negatively correlated with the monthly family incomes of patients. The scores of men were lower than those of women. Medical staff are prompted to pay more attention to female patients, patients with less monthly family incomes and prolonged disease duration, take active intervention measures to decrease the patients’ illness uncertainty, guide the patients to have a firm conviction so that they have a positive attitude to face up to the disease and be cooperative in the treatments, and improve treatment compliance.

### 6.1 Limitations

As with any study, this one has some limitations. In this study, only the self‐rating scale was used to investigate the illness uncertainty of patients with COVID‐19 treated in a mobile cabin hospital was investigated. Cultural differences in epidemic prevention and control exist in different regions (Wang, Chudzicka‐Czupała, et al., 2020), which might influence the representativeness of the sample and the generalizability of the result. Another issue is that, due to the nature of cross‐sectional studies, this study did not carry out further research on the dynamic changes of illness uncertainty and the long‐term impact on patients. A study has shown that there were no significant longitudinal changes in stress, anxiety and depression levels in the general population after 4 weeks (Wang, Chudzicka‐Czupała, et al., 2020; Wang et al., 2020b). A further longitudinal design is needed to explore illness at different stages of disease and its impact on patients.

## CONFLICT OF INTEREST

The authors declare that they have no conflict of interest.

## AUTHOR CONTRIBUTIONS

Made substantial contributions to conception and design, or acquisition of data, or analysis and interpretation of data; D. L., C. L. Involved in drafting the manuscript or revising it critically for important intellectual content; D. L., C. L., D. S. Given final approval of the version to be published. Each author should have participated sufficiently in the work to take public responsibility for appropriate portions of the content; D. L., C. L., D. S. Agreed to be accountable for all aspects of the work in ensuring that questions related to the accuracy or integrity of any part of the work are appropriately investigated and resolved; D. S.

## Data Availability

Data available on request from the authors.
